# Quality of Life Among Kurdish Patients With Knee Osteoarthritis in Duhok Governorate

**DOI:** 10.7759/cureus.73610

**Published:** 2024-11-13

**Authors:** Havrest N Sadiq, Mohammed T Rasool

**Affiliations:** 1 Department of Internal Medicine, College of Medicine, University of Duhok, Duhok, IRQ

**Keywords:** 36-item short form survey, duhok, iraq, knee, knee osteoarthritis/ koa, kurdistan region of iraq, qol, quality of life (qol), sf-36

## Abstract

Background: Osteoarthritis (OA) is a chronic disease and a leading cause for pain, stiffness, functional dependence, and diminished quality of life (QoL). The assessment of the latter in OA is becoming increasingly common in both research and clinical settings.

Objectives: The study aimed to examine OA among Kurdish patients with unilateral or bilateral OA.

Methods: A cross-sectional study was conducted at the Duhok Centre of Rheumatology and Medical Rehabilitation in the Kurdistan Region of Iraq, involving 262 patients aged 40-70 who have unilateral or bilateral OA.

Results: The study subjects had the lowest mean QoL score in the domain of role limitations due to physical health problems (14.22±34.124). The highest mean QoL score was in the social functioning domain (86.78±20.878). Females had a statistically significant lower QoL mean score across most domains, except for emotional role functioning. Older patients (over 50) had lower scores in several domains, such as physical function, physical role functioning, energy/fatigue, social functioning, and bodily pain. Longer disease duration is correlated with lower QoL scores in most domains, except emotional well-being. Obesity negatively impacted QoL in terms of physical function and pain dimensions. Finally, the severity of osteoarthritis is positively correlated with a lower QoL score in the domains of physical functioning, physical role functioning, energy/fatigue, social functioning, and the pain component.

Conclusion: QoL is diminished in Kurdish patients with unilateral or bilateral knee OA and is influenced by age, gender, obesity, disease duration, and OA severity.

## Introduction

Osteoarthritis (OA) is the most prevalent chronic joint disorder, the leading cause of joint pain and disability. The knee is the most clinically significant site affected by OA. Globally, 7.6% of the population is affected by osteoarthritis. OA was ranked as the 14th most common cause of age-standardized years lived with disability (YLDs) and ranked as the seventh cause of YLDs among individuals aged 70 years or older in 2020 [1].

Chronic disorders, such as OA, frequently exert a significant impact on functionality and QoL. It is essential to recognize that mere survival is no longer viewed as the sole objective; the aim is to enhance, restore, or maintain QoL [2].

Although the World Health Organization (WHO) defines health as “a state of complete physical, mental and social well-being and not merely the absence of disease or infirmity”, there is no consistent method for defining the QoL concept [3]. QoL is a broad notion that is affected by physical health, psychological conditions, and social relationships [4]. WHO defines QoL as an individual’s impression of their status in life considering the cultural and value system in which they exist, and in relation to their goals, expectations, standards, and concerns [5]. This instrument is reliable with a Cronbach's alpha greater than 0.85 [6].

There are many instruments to measure QoL such as the Medical Outcomes Study 36-Item Short Form (SF-36) Health Survey, EuroQol Instrument (EQ-5D), the Quality of Well-Being (QWB) Scale, Nottingham Health Profile (NHP), the Dartmouth Primary care Cooperative Information Project (COOP) Charts, Visual Analogue Scale EQ-VAS, Patient-Reported Outcomes Measurement Information System (PROMIS), and Health Utilities Index (HUI) [3,7]. However, the SF-36 instrument is the most commonly used tool in evaluating the QoL [8].

Understanding QOL is crucial for understanding the implication of diseases and treatment decision-making approaches across different age and cultural groups. A substantial quantity of published research on QoL in patients with chronic diseases, including OA, exists worldwide; however, most of these studies were conducted in developed countries. Since the QoL concept varies across cultures, there is a paucity of knowledge about QoL status in different health conditions in developing countries [9]. To our knowledge, there is a lack of research on QoL among patients with knee OA in developing countries, especially in Iraq.

This study aims to assess the QoL among Kurdish patients with knee osteoarthritis by evaluating various dimensions of QoL, including physical functioning, emotional well-being, and social interactions. Additionally, we aim to identify the demographic and clinical factors that significantly impact QoL in this population, such as age, gender, disease severity, and duration of osteoarthritis.

## Materials and methods

Study design and setting

A cross-sectional study was conducted at the Duhok Centre for Rheumatology and Medical Rehabilitation from August 2023 to April 2024. This center is the only referral center in the Duhok governorate in the Kurdistan Region of Iraq.

Study population

The inclusion criteria included individuals who had a diagnosis of unilateral or bilateral knee osteoarthritis and were between the ages of 40 and 70, irrespective of gender.

We used a plain radiograph (anteroposterior and lateral views) to confirm and measure the stage of OA articular changes. This imaging technique is still considered the most commonly used modality to confirm a structural diagnosis of OA [10].

The exclusion criteria encompassed individuals with medical conditions such as kidney, liver, cardiac, neurological diseases, cognitive impairment, malignancies, previous knee surgeries, or other diseases related to the osteoarticular system, such as rheumatic or metabolic bone diseases that could adversely impact the patient's quality of life and functional independence.

Eligible patients, whether newly or previously diagnosed, who visited the center were invited consecutively to participate in the study until the required sample was reached. All of them agreed to participate in the study.

Sampling method and sample size

A convenient sampling method to choose the eligible patients was used until the minimum required sample was obtained. The following sample size calculation was used in estimating the minimum required sample size [11].

n= Z2 P (1- P)/d2

In our study, we used a Z-value of 1.96, which corresponds to 95% confidence intervals, a p-value of 0.37, which represents the expected proportion of Knee OA patients having functional limitations based on published literature [12], and a precision (d) value of 0.06, which is less than one-fourth of the expected p-value as recommended in the literature [13]. Therefore, the estimated minimum sample size was approximately 250 patients.

Data collection tool

The researchers collected data using a structured questionnaire. The background information encompassed details such as gender, age, occupation, educational level, height and weight, the presence of comorbid diseases, the number of years since diagnosis, and the grade of osteoarthritis (OA) based on the Kellgren and Lawrence (KL) grading system which had a moderate to very good reliability. On knee plain radiographs, the KL system classifies the severity of the OA into five grades, from zero (indicating no OA) to 4 (indicating severe OA) [14].

QoL is assessed using questions highlighted in the Short-Form Health Survey (SF-36) tool. This tool comprises 36 items grouped into eight main domains: physical functioning (10 items), role limitation due to physical health problems (4 items), role limitations due to emotional problems (3 items), Energy/fatigue (4 items), emotional well-being (5 items), social functioning (2 items), bodily pain (2 items), and general health perception (5 items) [15].

The score of each item and domain was converted to a scale of values that ranged from 0 (corresponding to worst health status) to 100 (corresponding to perfect health status). A mean score of 50 has been expressed as a normative cut-off value for all items and domains [16,17].

Ethical considerations

The study was approved by the Duhok Research Ethics Committee (Reference No. 26072023-6-12 on 26 July 2023).

Data analysis

Data processing and processing were done using IBM Corp. Released 2016. IBM SPSS Statistics for Windows, Version 24.0. Armonk, NY: IBM Corp. Quantitative data were presented as mean and standard deviation (SD). Categorical data were summarized using count and percentage. Independent samples t-test and ANOVA test were used to test the difference in mean QoL score between two groups, and among three groups or more, respectively. Statistically significant differences in the ANOVA test underwent post-hoc comparisons using the Bonferroni test. The level of significance was set at 0.05. A p-value of 0.05 or less was considered statistically significant.

## Results

Table 1 shows that 56.5% of participants aged more than 50 years and 67.6% were females. Moreover, 60.3% were obese and 34.0% were overweight. Eighty-one (30.9%) of the participants had been diagnosed with OA for more than 5 years. Among the enrolled participants, 45% had grade II OA, followed by grade I (27.1%), grade III (22.5%), and grade IV (5.3%). Furthermore, 40.8% of the patients presented with other comorbidities.

**Table 1:** Distribution of the study participants according to selected background characteristics (N = 262)

Characteristics		No. (%)
Age		
	50 or less	114 (43.5)
	More than 50	148 (56.5)
Gender		
	Male	85 (32.4)
	Female	177 (67.6)
Educational level		
	Illiterate	101 (38.5)
	Can read and write	52 (19.8)
	Primary	45 (17.2)
	Secondary	28 (10.7)
	Preparatory	5 (1.9)
Occupation		
	Jobless	26 (9.9)
	Housewife	149 (56.9)
	Administrative (employee)	37 (14.1)
	Service worker	50 (19.1)
Body mass index		
	Normal weight	15 (5.7)
	Overweight	89 (34.0)
	Obesity	158 (60.3)
Years since diagnosis		
	≤ 5 years	181 (69.1)
	> 5 years	81 (30.9)
Radiological grading of osteoarthritis		
	Grade I	71 (27.1)
	Grade II	118 (45.0)
	Grade III	59 (22.5)
	Grade IV	14 (5.3)
Presence of comorbidities		
	Yes	107 (40.8)
	No	155 (59.2)

Table 2 presents the mean and standard deviation of the QoL score of each of the SF-36 domains. The lowest mean score was in the domain of role limitations due to physical health problems (14.22±34.124), and the highest mean score was in the social functioning domain (86.78±20.878).

**Table 2:** Quality of life mean score and standard deviation of the study participants (N = 262)

SF-36 domain	Mean (SD)
Physical functioning	46.28 (28.28)
Role limitations due to physical health problems	14.22 (34.12)
Role limitations due to emotional problems	78.24 (41.13)
Energy/fatigue	59.03 (15.77)
Emotional well-being	60.49 (15.96)
Social functioning	86.78 (20.87)
Pain	48.44 (19.62)
General health	61.18 12.32)

Table 3 presents the average QoL score across the eight SF36 variables according to specified background parameters. There was a statistically significant difference in the mean scores between males and females across most SF-36 domains, apart from emotional role functioning. Female has a lower score in physical functioning (p< 0.001), physical role functioning (p = 0.002), energy/fatigue (p<0.001), emotional well-being (p<0.001), social functioning (p<0.001), and pain (p<0.001) dimensions. Participants who aged more than 50 years had significantly lower mean scores for physical function (p<0.001), physical role functioning (p<0.001), energy/fatigue (p = 0.010), social functioning (p = 0.028), and pain (p<0.001) compared to those who are 50 years or less.

**Table 3:** Dimensions of the SF36 by selected background characteristics

Characteristics	N	Physical functioning			Role functioning/physical			Role functioning/emotional			Energy/fatigue			Emotional well-being			Social functioning			Pain			General health		
		Mean (SD)	Statistics value*	p-value	Mean (SD)	Statistics value*	p-value	Mean (SD)	Statistics value*	p-value	Mean (SD)	Statistics value*	p-value	Mean (SD)	Statistics value*	p-value	Mean (SD)	Statistics value*	p-value	Mean (SD)	Statistics value*	p-value	Mean (SD)	Statistics value*	p-value
Gender																									
Male	85	59.94 (25.66)	5.740	<0.001	25.00 (42.78)	3.137	0.002	84.71 (36.20)	1.880	0.062	67.47 (14.07)	6.595	<0.001	68.00 (13.03)	5.987	<0.001	93.53 (14.76)	4.278	<0.001	55.26 (18.94)	4.009	<0.001	55.65 (13.62)	-4.870	<0.001
Female	177	39.72 (27.17)			9.04 (27.75)			75.14 (43.05)			54.97 (14.94)			56.88 (16.01)			83.55 (22.58)			45.17 (19.14)			63.84 (10.71)		
Age																									
50 or less	114	60.39 (25.69)	7.875	<0.001	25.66 (42.65)	4.614	<0.001	75.15 (43.18)	-1.058	0.291	61.89 (15.08)	2.604	0.010	59.05 (16.18)	-1.279	0.202	89.91 (17.82)	2.210	0.028	53.90 (19.31)	4.067	<0.001	60.18 (12.72)	-1.163	0.246
More than 50	148	35.41 (25.28)			5.41 (22.12)			80.63 (39.46)			56.82 (15.98)			61.59 (15.75)			84.38 (22.72)			44.24 (18.87)			61.96 (11.99)		
BMI index																									
Normal weight	15	64.67 (17.16)	12.734	<0.001	26.67 (45.77)	2.008	0.136	86.67 (35.18)	0.404	0.669	66.00 (12.98)	2.075	0.128	62.13 (13.84)	2.541	0.081	89.17 (22.09)	1.258	0.286	57.00 (21.88)	6.544	0.002	59.00 (13.78)	1.262	0.285
Overweight	89	54.94 (28.53)			17.42 (36.61)			76.40 (42.70)			59.94 (16.96)			63.37 (15.15)			89.33 (18.80)			53.06 (19.44)			59.83 (12.41)		
Obesity	158	39.65 (26.98)			11.23 (31.10)			78.48 (40.88)			57.85 (15.18)			58.71 (16.42)			85.13 (21.81)			45.03 (18.83)			62.15 (12.10)		
Years since diagnosis																									
≤ 5 years	181	51.55 (27.58)	4.684	<0.001	18.51 (37.95)	3.822	<0.001	80.66 (39.60)	1.368	0.174	60.99 (15.35)	3.026	0.003	61.88 (15.24)	2.120	0.045	89.36 (19.20)	2.822	0.006	51.71 (18.92)	4.152	<0.001	60.47 (12.73)	-1.471	0.143
> 5 years	81	34.51 (26.35)			4.63 (20.58)			72.84 (44.13)			54.63 (15.90)			57.38 (17.16)			81.02 (23.31)			41.14 (19.30)			62.78 (11.26)		
Grading of OA																									
Grade I	71	67.61 (23.26)	37.303	<0.001	35.56 (46.41)	15.836	<0.001	81.69 (38.95)	1.659	0.176	65.42 (14.65)	8.441	<0.001	61.52 (15.16)	0.641	0.589	93.49 (13.83)	9.778	<0.001	59.51 (18.49)	25.261	<0.001	59.44 (12.86)	1.166	0.323
Grade II	118	45.42 (26.26)			9.32 (28.64)			81.64 (38.63)			59.03 (15.03)			60.71 (15.56)			88.24 (18.93)			49.05 (17.46)			61.10 (13.01)		
Grade III	59	30.25 (20.77)			1.69 (13.01)			70.62 (45.52)			53.31 (15.80)			60.07 (17.33)			80.72 (23.70)			40.34 (16.87)			62.54 (10.96)		
Grade IV	14	12.86 (12.35)			0.00 (0.00)			64.29 (49.72)			50.71 (15.54)			55.14 (17.90)			66.07 (33.04)			21.43 (11.03)			65.00 (7.59)		
Presence of comorbidities																									
Yes	107	31.87 (23.40)	-7.551	<0.001	3.97 (18.22)	-4.702	<0.001	79.44 (40.08)	0.390	0.697	54.21 (14.31)	-4.243	<0.001	59.63 (14.78)	-0.726	0.469	82.94 (22.99)	-2.498	0.017	41.33 (19.09)	-5.102	<0.001	64.95 (9.89)	4.247	<0.001
No	155	56.23 (27.10)			21.29 (40.27)			77.42 (41.94)			62.35 (15.91)			61.08 (16.75)			89.44 (18.90)			53.35 (18.50)			58.58 (13.16)		
* T-test value using independent samples t-test for gender, age, years since diagnosis, presence of comorbidities variables; and F-statistics using ANOVA for BMI index and grading of OA variables																									

As the duration since diagnosis increased, the average QoL score diminished across the majority of domains. Patients diagnosed over five years ago exhibited markedly diminished mean quality of life in the physical functioning dimension (p<0.001), physical role functioning dimension (p<0.001), energy/fatigue dimension (p=0.003), social functioning dimension (p=0.006), and pain dimension (p<0.001). Patients with the diseases for more than five years showed a slightly lower mean QoL score on the emotional well-being dimension (p = 0.045), but there was no significant difference in the mean QoL score on the emotional role functioning and general health dimensions (p = 0.174 and 0.143, respectively).

Obesity impacts the QoL mean score for physical function and pain dimensions. There was a statistically significant difference in the mean QoL score on the physical functioning dimension across the different BMI groups (p<0.001) and pain dimension (0.002). Further, post-hoc comparison, as shown in Table 4, indicated there was a statistically significant difference in the mean score on the physical functioning dimension between normally weighed patients and obese patients (p = 0.002) and between overweight patients and obese patients (p<0.001). Moreover, an observed difference in the average QoL score for the pain dimension was seen between overweight and obese patients (p = 0.005). Obese patients exhibited worse mean OoL scores than both overweight and normal-weight patients.

**Table 4:** Post-hoc comparisons of the mean QoL score by BMI index using Bonferroni test

Dimension	Groups of BMI index	Mean difference in QoL score	p-value
Physical functioning	Normal weight vs. Overweight	9.723	0.599
	Normal weight vs. Obesity	25.015	0.002
	Overweight vs. Obesity	15.292	<0.001
Pain	Normal weight vs. Overweight	3.938	1.000
	Normal weight vs. Obesity	11.968	0.066
	Overweight vs. Obesity	8.030	0.005

A statistically significant difference was also seen in the QoL mean score for physical functioning, physical role functioning, energy/fatigue, social functioning, and pain component (p<0.001 for all dimensions) among the different grades of OA. The higher the grade, the lower the QoL mean score. Further post-hoc analysis, as depicted in Table 5, indicated that, in general, the higher the OA grade, the lower the QoL mean score. There was a statistically significant difference in the QoL on physical functioning dimension between grade I and other grades (II, III, IV) (p<0.001) and between grade II and other higher grades (III and IV) (p<0.001). On the physical role limitation dimension, there was a statistically significant difference in the mean QoL between grade 1 and the other three grades (p<0.001 for grades II and III and p=0.001 for grade IV). On energy/fatigue dimensions, the statistically significant difference was between grades I and II (p = 0.032), between grades I and III (<0.001), and between grades I and IV (p = 0.006). On the social functioning dimension, there was a statistically significant difference between grade I and grade III (p = 0.002), grade I and IV (p<0.001), and grade II and IV (0.001). On the pain dimension, the QoL mean score was significantly different between grade and the other 3 grades (p<0.001 for the comparisons), between grades II and III (p=0.011) and grades II and IV (p<0.001), and between grade III and grade IV (p=0.002).

**Table 5:** Post-hoc comparisons of the mean QoL score by OA grading using Bonferroni test

Dimension	Groups of OA gradings	Mean difference in QoL score	p-value
Physical functioning	Grade I vs. Grade II	22.182	<0.001
	Grade I vs. Grade III	37.351	<0.001
	Grade I vs. Grade IV	54.748	<0.001
	Grade II vs. Grade III	15.169	<0.001
	Grade II vs. Grade IV	32.567	<0.001
	Grade III vs. Grade IV	17.397	0.087
Role limitation/physical	Grade I vs. Grade II	26.241	<0.001
	Grade I vs. Grade III	33.868	<0.001
	Grade I vs. Grade IV	35.563	0.001
	Grade II vs. Grade III	7.627	0.784
	Grade II vs. Grade IV	9.322	1.000
	Grade III vs. Grade IV	1.695	1.000
Energy/fatigue	Grade I vs. Grade II	6.397	0.032
	Grade I vs. Grade III	12.117	<0.001
	Grade I vs. Grade IV	14.708	0.006
	Grade II vs. Grade III	5.720	0.111
	Grade II vs. Grade IV	8.311	0.319
	Grade III vs. Grade IV	2.591	1.000
Social functioning	Grade I vs. Grade II	5.244	0.483
	Grade I vs. Grade III	12.766	0.002
	Grade I vs. Grade IV	27.414	<0.001
	Grade II vs. Grade III	7.521	0.111
	Grade II vs. Grade IV	22.170	0.001
	Grade III vs. Grade IV	14.649	0.083
Pain	Grade I vs. Grade II	10.460	<0.001
	Grade I vs. Grade III	19.168	<0.001
	Grade I vs. Grade IV	38.078	<0.001
	Grade II vs. Grade III	8.708	0.011
	Grade II vs. Grade IV	27.618	<0.001
	Grade III vs. Grade IV	18.910	0.002

## Discussion

The SF-36 instrument provides measures of patient health status across eight domains and has been widely used in rheumatology research. Our research is the first study assessing the quality of life among Kurdish patients with knee OA. The main findings of this study are that patients with OA exhibited a significant negative impact on QoL. We recruited 262 patients aged 40-70 years who had a diagnosis of unilateral or bilateral knee OA. Among all the eight domains, the role limitation due to physical health problems QoL domain score was the lowest and the social functioning QoL domain score was the highest. Other studies also indicated that the emotional and social domains of QoL were less affected compared to the physical domains of the QoL [12,18].

Findings from this study indicate that females have, in general, a lower quality of life compared to males. Emotional role functioning is an exception. These results corroborate a previous systematic analysis showing that females have much higher pain, experience more physical health issues, and show more knee functional dependence [19]. The latter study also indicates that depression, anxiety, and social support did not differ between males and females. Another cross-sectional also indicated gender had a significant relationship with most of the SF-36 domains except for role limitations due to emotional problems and social functioning [18]. Moreover, increasing age was another predictor for lower QoL scores in five domains (physical function, physical role functioning, energy/fatigue, social functioning, and pain). Age is commonly associated with lower SF domain QoL scores [20].

Longer duration of OA was a significant predictor of the lower QoL on physical and social domains but had less impact on the emotional role functioning. Shalhoub et. al [21] also indicated that a shorter duration of patient symptoms was associated with higher health-related QoL.

Obesity (BMI of 30 or more) had a negative impact on the QoL score for physical function and pain dimensions but had no effect on social, emotional, and mental domains. The latter is not consistent with other studies. A study in Brazil found that higher BMI negatively impacted the physical, social, and mental aspects of QoL [22]. The Brazilian study used the WHO QoL instrument to measure QoL. The difference in instrument and cultural context is a plausible reason for the difference in the result of our study with the Brazilian study. Another study also found that obesity has a negative impact on the QoL but the impact was minimal when overweighed and normally weighed patients were compared [23].

Finally, the mean QoL score was lower with an increasing grade of OA. This was more evident in the physical functioning, physical role functioning, energy/fatigue, social functioning, and pain domains. Evidence in the literature also indicates that the severity of the OA is a strong predictor of lower QoL [8,24]. This high level of correlation between QoL and grading of OA is possibly due to the increasing symptoms such as pain which limits the activities of patients with knee OA [21].

This study is the first to assess the quality of life among Kurdish patients with knee osteoarthritis, utilizing a relatively large sample size of 262 participants. The use of the SF-36, a validated instrument for measuring QoL across multiple domains, adds rigor to our findings. Additionally, the focus on this specific demographic and cultural context provides valuable insights that can inform future research and healthcare interventions in the region. Despite these strengths, our study has several limitations. The convenience sampling method employed may introduce bias, limiting the generalizability of our findings to the broader Kurdish population. Conducting the study in a single rheumatology center may not accurately reflect the experiences of all Kurdish patients with knee OA. Furthermore, potential confounding factors, such as socioeconomic status and access to healthcare, were not controlled for in our analysis. Lastly, cultural factors may influence perceptions of QoL, which could affect how respondents interpret and answer the SF-36 questions.

## Conclusions

Overall, the findings of this study show that Kurdish patients with knee OA are not much different from other socio-cultural contexts. Osteoarthritis has a substantial impact on quality of life. Physicians shall do regular assessments of the quality of life in patients with knee osteoarthritis to ensure they receive the most appropriate treatment protocol that makes them better physically, mentally, and socially. A community-based cross-sectional study is recommended to be conducted to assess QoL among OA patients.
